# Osmotin: A Cationic Protein Leads to Improve Biotic and Abiotic Stress Tolerance in Plants

**DOI:** 10.3390/plants9080992

**Published:** 2020-08-04

**Authors:** Muhammad Ajmal Bashir, Cristian Silvestri, Touqeer Ahmad, Ishfaq Ahmad Hafiz, Nadeem Akhtar Abbasi, Ayesha Manzoor, Valerio Cristofori, Eddo Rugini

**Affiliations:** 1 Department of Horticulture, PMAS Arid Agriculture University, Rawalpindi 46300, Pakistan; muhammadajmal@unitus.it (M.A.B.); touqeer@uaar.edu.pk (T.A.); drishfaq@uaar.edu.pk (I.A.H.); nadeem.abbasi@uaar.edu.pk (N.A.A.); 2 Department of Agriculture and Forest Sciences (DAFNE), University of Tuscia, 01100 Viterbo, Italy; valerio75@unitus.it (V.C.); rugini@unitus.it (E.R.); 3 Barani Agricultural Research Institute, Chakwal 48800, Pakistan; manzoorayesha12@yahoo.com

**Keywords:** antifungal activity, drought, proline, salinity, tobacco osmotin gene

## Abstract

Research on biologically active compounds has been increased in order to improve plant protection against various environmental stresses. Among natural sources, plants are the fundamental material for studying these bioactive compounds as their immune system consists of many peptides, proteins, and hormones. Osmotin is a multifunctional stress-responsive protein belonging to pathogenesis-related 5 (PR-5) defense-related protein family, which is involved in inducing osmo-tolerance in plants. In this scenario, the accumulation of osmotin initiates abiotic and biotic signal transductions. These proteins work as antifungal agents against a broad range of fungal species by increasing plasma membrane permeability and dissipating the membrane potential of infecting fungi. Therefore, overexpression of tobacco osmotin protein in transgenic plants protects them from different stresses by reducing reactive oxygen species (ROS) production, limiting lipid peroxidation, initiating programmed cell death (PCD), and increasing proline content and scavenging enzyme activity. Other than osmotin, its homologous proteins, osmotin-like proteins (OLPs), also have dual function in plant defense against osmotic stress and have strong antifungal activity.

## 1. Introduction

Plants come across various biotic and abiotic stresses during their crop cycle due to constantly changing environmental conditions that lead to different morphological, physiological, and biochemical changes, resulting in severe effects on their growth, production, and even survival [1]. Therefore, a better comprehension of the response and adaptation of plants to global changes is urgently needed in a climate changing scenario [2]. Biotic factors that provoke stress conditions are fungi, viruses, and bacteria, whereas abiotic factors consist of high or low temperature, salinity, drought, hypoxia, pollution, and ultraviolet radiations. To combat these stresses, plants initiate their defense mechanisms that include structural responses such as strengthening of cell wall and development of waxy epidermal cuticles, etc. All these responses provide plants with enough strength and rigidity to reduce the effect of injuries caused by both biotic and abiotic stresses [3]. Another important stress mechanism activated by plants is metabolic alterations, which involve synthesis of many proteins, particularly pathogenesis-related (PR) proteins [4]. These proteins belong to a super large family of defense proteins in plants, have antimicrobial properties under in vitro conditions, and their overexpression induces stress tolerance in plants. PR proteins are divided into seventeen families on the basis of their structure, mode of action, and amino acid sequence. In fact, proteins belonging to the PR-5 family are important with respect to stress tolerance [3,5]. The PR-5 proteins are cysteine-rich, and are also known as thiamine-like proteins (TLPs) due to similar sequence homology with thiamine proteins extracted from arils of the sweet prayer plant (*Thaumatococcus danielli*) [6,7]. In fact, osmotin and osmotin-like proteins (OLPs) from the PR-5 family are recognized as most important for their osmo-protectant role and antifungal properties [8].

## 2. Osmotin

Osmotin is a multifunctional protein whose overexpression induces abiotic stress tolerance and provides protection from fungal infections as well. It also has anti-bacterial activity against many food-borne pathogens [3]. It was discovered first in tobacco plants (*Nicotiana tabacum* var. Wisconsin 38) that showed tolerance to salt stress [9]. Later, some related proteins (homologues) were found in the whole plant kingdom among different monocot and dicot plants as well [10]. In its pre-protein form, osmotin is synthesized in vacuoles and keeps molecular weight of 26.4 kDa, whereas in its mature form the molecular weight is reduced to 24 kDa. This cysteine-rich protein provides protection to the cell membrane under low water potential and it also has a significant role in osmoregulation and food preservations [3].

Osmotin’s name is derived from its function to lower osmotic potential under stress. Osmotin and its homologous proteins (osmotin-like proteins (OLPs)) are naturally present in all fruits and vegetables and induce stress tolerance in them [11]. Its expression has also been observed in different plant organs such as leaves, trichomes, tobacco flowers, olive seed endosperm and coat, somatic embryos, and grape skin. It was found in tissues such as stem and root epidermis and also found in corolla but in less quantity. However, in some tissues, its concentration is so minute that it is untraceable [3].

Osmotin and osmotin-like proteins have tissue-specific activities with NaCl, ethylene, and abscisic acid (ABA) treatments, and under oxidative stress they are expressed in dormant regions of the root apex and meristematic regions of the shoot apex, respectively [12]. This protein has been also induced in tobacco roots and cultured cells in response to abscisic acid treatments. In addition, the transcription of osmotin is induced by ABA in plants and its transcript level with the increase in the endogenous ABA level; however, low water potential is essential for its accumulation in the cells. Moreover, its expression is controlled by various hormonal and environmental signals (wounding, drought, salinity, tobacco mosaic virus infection, ethylene, and ABA), complex development, and tissue-specific mechanisms that cause transcriptional and post-transcriptional regulation of osmotin mRNA [13,14].

Ethylene-induced accumulation of osmotin is fundamental in young leaf tissues and roots. Accumulation of osmotin becomes stable after removing NaCl from the environment and can last up to forty generations [13]. Therefore, accumulation of osmotin protein in cultured cells of tobacco subjected to salt stress (428 mM) accounts for 10–12% of total cell protein [11,15], whereas untreated cell cultures (no NaCl) also showed expression of osmotin which indicates that at basal level it acts as a housekeeping gene [16].

### 2.1. Structure

Osmotin is a group of cationic proteins that exists in two forms, one having an isoelectric point (pI) of 7.8 and the other with pI greater than 8.2. They slightly differ in molecular weight and result in water soluble osmotin I and water insoluble osmotin II [13]. Osmotin and thiamine both have similar molecular weight, structure, disulfide bonds, basic pI proteins, proline percentage, and absence of sulfhydryl residues, but thiamine is sweet in taste as compared to osmotin [3], due to absence of lysine residues that are found in thiamine proteins [15]. In addition, grooves on osmotin proteins contain strong amino acid residues (Asp4, Asp, Glu), that lead to its acidic nature because of the negative charge but osmotin-like proteins (homologous proteins) are neutral in nature [17].

In fact, osmotin also has a peptide sequence that is similar to potato PR protein C, tobacco antiviral protein gp 22, tomato NP protein 24, barley thiamine-like proteins, and the maize trypsin/a-amylase inhibitor [18].

### 2.2. Function

**Figure 1:**
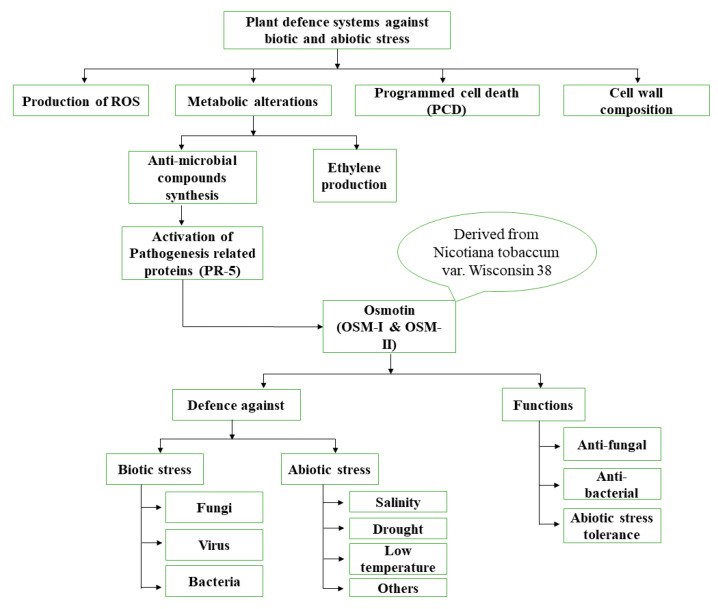
Schematic representation of the role of osmotin in plant defense mechanisms.

Osmotin accumulates in plants under both stressed and normal conditions (Figure 1). Under no stress conditions, osmotin acts as a housekeeping gene involved in basic cell metabolism [17]. It is a multidomain protein and each domain performs its own particular function independently or synergistically with other domains. One of the most important domains of osmotin is represented by kinase-like proteins. Protein kinases catalyze the phosphotransferase reaction that is crucial for signaling and regulatory processes in eukaryotic cells, such as assembly of macromolecules, enzyme activation, and protein localization and degradation [11].

Osmotin has an important role in plant immune systems during stress conditions [10]. It triggers defense mechanisms against stress in plants by working as a transcriptional regulator of genes encoding various stress-related enzymes or acts as signaling molecules through intracellular receptors. Moreover, osmotin also has a function to protect native proteins under stress conditions and to repair the denatured proteins as well [3].

Under abiotic stress conditions, it works as an osmoprotectant, by providing protection to enzymes and improve chaperone protein functions [10]. Moreover, it adjusts the osmotic potential in plants either through accumulation or compartmentation of solutes or through different structural and metabolic changes during osmotic adjustments [19].

## 3. Genetic Engineering

Previously, scientists have relied on identification of stress-tolerant genotypes for the development of stress-tolerant varieties, to be used for breeding through conventional means. However, conventional breeding methods for the development of stress-tolerant genotypes are not effective because of the availability of little genetic diversity and secondly the complex polygenic nature of the traits which also limits the successful production of tolerant genotypes [20]. Therefore, there is a need to develop modern biotechnological techniques that can produce novel varieties, which can survive to extreme stress conditions, continue their growth, and produce a higher yield under an unfavorable environment. Genetic engineering technology could be one of the main approaches to produce stress-tolerant crops [21,22].

Genetic modification has widely been used to improve indigenous crops by enhancing their tolerance against various biotic and abiotic stresses, therefore increasing their quantity and quality. It is one of the most effective and reliable techniques to introduce desirable genes in targeted plants [4]. Genetically modified plants have a fundamental importance in green biotechnology and development of transgenic crops with improved resistance against deadly disease and extreme environmental conditions, which play an important role in combating famine and drought at a global level [11]. Therefore, *Agrobacterium tumefaciens*-mediated transformation is one of the widely used methods for developing transgenic plants in many species [23].

### Agrobacterium Mediated Transformation

Gene transfer technology has become a useful alternative to speed up genetic improvement by correcting defects or conferring tolerance in well-known commercial varieties [24], and also speeding up the breeding programs, whereas the establishment of suitable novel genotypes by means of traditional crossbreeding requires a great deal of time [25]. Multiple genes have to be introduced for developing tolerance in plants against stress [26], such as osmotin, which plays an important role in improving various crop yields by protecting them from various biotic and abiotic stresses. This protein has been engineered to resolve many plant protection-related issues [12]. Therefore, genetic transformation of the osmotin gene is rather easy because its genome sequence analysis has confirmed that it has no introns [11].

## 4. Abiotic Stress Tolerance

### 4.1. Drought

Plants mainly require water and nutrients for their survival, growth, and development but, due to extreme climatic changes, agriculture is usually reliant on low resource environments. It is estimated that 30% of arable land will be lost by 2021 due to drought, and by 2050 this percentage could exceed 50% [27]. In fact, drought is the major factor that contributes towards food security issues. Scarcity of water is one of the main threatening issues to the agricultural world, as many countries cannot fulfill the water demand of growing crops. Furthermore, extreme changes in climate can lead to more severe and prolonged drought in many countries of the world [28].

Plant response towards stress belongs to a series of different events, including perception of stress, induction of signal transduction pathway, expression of stress-specific biomolecules, and imparting stress tolerance by synthesizing various metabolites and osmolytes. One such osmolyte is osmotin, which is isolated from tobacco cells and has been extensively used in the 1980s. Osmotin confers tolerance to plants by increasing their proline content. This role of osmotin is positively correlated with y-glutamine kinase and *γ*-pyrrolin-5-carboxylate reductase enzymes activity, which are accumulated by the plants under stress conditions to increase proline content. Therefore, proline degrading enzyme (proline dehydrogenase and proline oxidase) activity is also limited under stress conditions [29]. Accumulation of proline under different abiotic stresses is considered as an indication of stress tolerance [30], as an increase in proline content enables the plant to re-establish its osmotic homeostasis by increasing water potential and protecting important enzymes, proteins, and cellular organelles from abiotic stress-induced damages [31]. Osmotin expression under stress conditions induces 4–6 times more proline accumulation in cells [11]. According to [30], in transgenic calli of rubber trees (*Heveabrasiliensis*) there was a two-fold increase in proline accumulation in response to polyethylene glycol (PEG) treatment as compared to non-transgenic calli. Similarly, in transgenic cotton (*Gossypium hirsutum*) [14] and olive (*Olea europea*) [27] plants, tobacco osmotin not only showed proline accumulation and antioxidant enzymes activity but also reduced electrolyte leakage, lipid peroxidation, and hydrogen peroxide production. In carrot (*Daucus carota*), transgenic plants have depicted a slower rate of wilting as compared to wild type plants and showed a faster recovery rate when drought stress level was increased [28]. In transgenic tomato (*Solanum lycopersicum*) plants, various morphological and physiological changes such as leaf expansion and higher water and chlorophyll content indicate plant resistance mechanisms against drought stress [32].

The effects of osmotin on somatic embryos of tea (*Camellia sinensis*) has been studied by [15], which describes that transformed embryos showed an increase in storage reserve with improved germination percentage and maturation rate. Furthermore, when somatic embryos were desiccated, they still germinated up to 40% in comparison to control embryos which became necrotic and failed to germinate. Similarly, in transgenic tea plants, when exposed to PEG-induced water stress, they not only increased activity of reactive oxygen species (ROS) scavenging enzymes but also increased the level of caffeine and flavan-3-ols, an important tea quality trait [33].

In addition, under stress condition, osmotin interacts with other proteins to induce signal flux that enhances the expression of defensive genes by changing the balance between cell death and protein survival [25]. Most of the osmotin protein is localized in the electron-dense bodies of vacuoles and a minute amount is present in cytoplasm which has been detected in barley [28].

### 4.2. Salinity

Salinity is one of the most important stresses that poses a global threat to all agricultural commodity and significantly affects crop growth and yield and can reduce world agricultural productivity up to 70% [18]. Saline soils have a high level of sodium and chloride that exert a stress on plants. In fact, both saline (397 Mha) and sodic (434 Mha) soils have affected about 800 Mha of land globally. Salinity not only inflicts ion toxicity (Cl^−^, Na^+^, SO_4_^2−^ = SO_4_^2+^) and nutrient deficiency (N, P, K, Ca, Zn, and Fe) on plants but also induces oxidative and osmotic stress in plants [31].

Salt stress affects different metabolic activities in plants by activating or inhibiting certain enzymes which ultimately results in reduction of water potential, CO_2_ assimilation, ion imbalance or alteration in accumulation of different osmotically active compounds, and synthesis of various proteins [34]. Moreover, it also induces certain biochemical and physiological changes such as stomatal closure, reduced photosynthetic rate, increased respiration, suppressed cell growth, and increased accumulation of reactive oxygen species (ROS). These ROS at higher concentrations can damage various macromolecules such as membrane lipids, proteins, and nucleic acids. Salinity also affects the plant tissues in different ways, such as water deficiency in cells due to higher solute concentration in soil, and induces ion-specific stress due to altered Na^+^, Cl^−^ concentration, and K^+^/Na^+^ ratio that interfere with the nutrient uptake. Hence, higher accumulation of salt ions has toxic effects on cells; therefore, they must be separated from cell machinery, which can be achieved by compartmentation of solutes. In order to overcome salt stress severity, plants evolve different biochemical mechanisms, that include salt ion exclusion, control uptake of ions by roots and their transportation to different plant organs and tissues, ions compartmentalization, changes in photosynthesis pathways and membrane structure, induction of compatible osmolytes, antioxidant enzymes, and phytohormones. In addition, the plants also develop molecular mechanisms that consist of certain gene expression and accumulation of stress-related proteins such as osmotin [35], which results in detoxification, maintenance of cellular homeostasis, and growth recovery in plants under stress conditions [18].

As salt stress is mainly caused by Na^+^ toxicity, osmotin thus comes into play by protecting the cells from stress by sequestering Na^+^ and compartmentalizing them in intercellular spaces and vacuoles. Osmotin also controls the accumulation of hydrogen peroxide (H_2_O_2_), thus protecting the cells from damage caused by reactive oxygen species (ROS) [17]. Therefore, the level of osmotin in NaCl-adapted cells is fifteen times higher than in non-adapted cells [36]. In fact, the message for osmotin synthesis is constantly present in adapted cells during their growth phase; hence, it is decreased during the exponential phase and continues to increase when the plant enters its stationary phase [37].

A study has been conducted by [29] in order to develop salinity tolerance in Indica rice (*Oryza sativa*) through genetic transformation. For this purpose, tobacco osmotin was transferred in rice calli through *Agrobacterium tumefaciens* and acclimatized under in vivo conditions. Results showed that transgenic plants under NaCl stress had the highest proline level, which results in increased chlorophyll content, higher ribulose oxygenase activity, and lower ROS production. Furthermore, these plants survived until harvesting and produced fully formed fertile seeds. In another study, an increase in proline accumulation due to the osmotin gene enabled transgenic tobacco explants to root rapidly even at a higher NaCl concentration (320 mM) under in vitro conditions [38]. Similarly, in transgenic plants of strawberry (*Fragaria × ananassa*) overexpression of the osmotin gene showed improved salinity tolerance due to an increase in total soluble protein concentration, chlorophyll content, and proline level [19]. Additionally, in barley (*Hordeum vulgare*), the osmotin gene-transformed plants had improved protein and chlorophyll content in response to salt stress. Moreover, transgenic plants also exhibit high ascorbate peroxidase (APX) activity with reduce lipid peroxidation [10].

In addition, biochemical analysis of chili (*Capsicum annum*) transgenic plants showed that overexpression of the tobacco osmotin gene improved the activities of antioxidant enzymes monodehydroascorbate reductase (MDHAR), dehydroascorbate reductase (DHAR), glutathione reductase (GR), superoxide dismutase (SOD), and ascorbate peroxidase (APX), increased levels of glycine betaine, proline, and chlorophyll content, and produced a 3.3 kg yield per plant. However, transgenic plants are similar to wild type plants [18]. In rubber tree (*Hevea brasiliensis*), transgenic embryonic calli under the lowest salt stress level (50 mM) effectively proliferated without any growth retardation, whereas control calli growth was severely reduced with a minimum survival rate (23%), while the transgenic calli still tolerated even the highest level of stress (150 mM) with a 72% survival rate [30]. Transgenic wheat plants also showed the ability to produce longer roots at extremely higher levels of salt stress (250 mM NaCl) [13].

### 4.3. Low Temperature

Freezing tolerance in plants depends upon the accumulation of cryoprotectants and biochemical and physical restructuring of cell membrane. These anatomical changes occur in specific tissues which enable plants to tolerate extracellular freezing. In most trees, the usual phenomena for tolerance against cold is the dormancy induction. Changes in Ca^2+^ have an important role in cold stress tolerance, as a gradual increase during low temperature indicates that the plant is adaptive to cold stress. Cold stress causes early cytoskeleton depolymerization and plasma membrane depolarization and changes are related to changes in Ca^2+^. On the other hand, programmed cell death (PCD) is also an important phenomenon for plant survival and growth. Changes in Ca^2+^ also induce programmed cell death [39]. Osmotin also has a role in cryoprotection against low temperature stress conditions. Under cold stress, osmotin was induced in endosperm and the seed coat of olive and OLPs were accumulated in *Solanum* pollens. It protects plants from cold by regulating alteration in cytoskeleton and improves calcium signaling [16]. The role of osmotin as a PCD inducer has been studied in yeast cells (*Saccharomyces cerevisiae*), as well in human-cultured cells [39].

Transgenic tomato (*Solanum lycopersicum*) plants expressing tobacco osmotin gene showed enhanced transcript expression of stress responsive genes ascorbate peroxidase (*APX*), pyrroline-5-carboxylate synthase (*P5CS*), and centromere binding factor 1 (*CBF1*) when exposed to cold stress. In addition, the metabolite analysis expressed an increase in ascorbate content and proline accumulation in transgenic plants [1]. Furthermore, these transgenic plants at reproductive stage also survived low temperature (+4 °C) without showing any visible chilling injuries, whereas wild type plants failed to recover at this temperature [20]. Tobacco osmotin in transgenic olive plants (*Olea europea*) confers cold tolerance by induced programmed cell death, and also prevents cytoskeleton depolymerization and blocks cold-induced calcium (Ca^2+^) signaling [39].

### 4.4. High Temperature

Cellular protein osmotin along with other stress-related proteins like late embryogenesis abundant protein, dehydrins, heat shock proteins, phosphatase, aquaporin, and protein kinases are expressed in plants in response to heat or high temperature stress [40]. During stress conditions, these proteins that are localized in the plasma membrane sense stress stimuli through intercellular compartments using calcium ions. These calcium ions further activate various signal pathways such as calcium-dependent protein kinase (CDPK) and mitogen-activated protein kinases (MAPK) to transduce different signals which initiate the transcription of many stress-related proteins in order to provide tolerance to heat stress [41]. In a study, a pretreatment heat shock treatment induced thermo-tolerance in germinating seeds of wheat (*Triticum aestivum*) by accumulating osmotin and heat shock proteins in plants [42]. Similarly, during repining of grapes, transcriptional expression of osmotin along with galactinol synthase and betaine-aldehyde dehydrogenase increased during high temperature in order to improve grape berries quality by increasing anthocyanin content and decreasing acidity in fruit [43,44].

## 5. Biotic Stress Tolerance

### 5.1. Fungal

Pest and pathogens can reduce plant yield up to 30% and among this loss percentage, 80% is attributed to fungal pathogens [45]. In response to microbial infection, plants initiate defense mechanisms that includes production of ROS, synthesis of secondary metabolites such as phytoalexins and deposition of mechanical barriers such as carbohydrates and hydroxyproline-rich glycoproteins, in order to limit the pathogen invasion [46]. A highly defensive reaction of plants against microbial attack is the hypersensitive response (HR). In this response plants induce cell death, therefore restricting microbes to the infection site because of localized rapid necrosis. The hypersensitive response is initiated when the plant resistance gene (*R*) recognizes an avirulent protein (*Avr*) secreted from the secretory systems of pathogens. However, cell death is not an effective measure to counter the attack of microbes because pathogens still extract nutrients from the dead cells. Thus, plants develop a new defense mechanism that involves the induction of pathogenesis related proteins (*PR-5*) [43].

**Figure 2:**
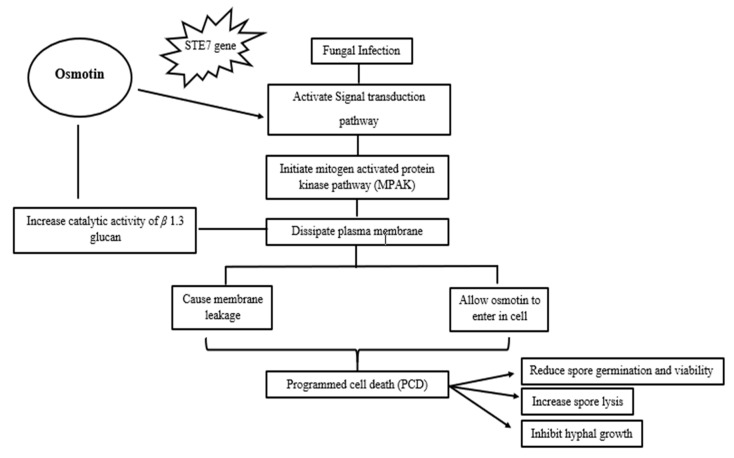
Schematic diagram representing the osmotin mechanism in inducing tolerance to fungal infections.

The PR-5 protein and osmotin acts as antifungal (Figure 2) by preventing spore germination, inhibiting hyphal growth, increasing spore lysis (hyphal tip lysis), and reducing spores’ viability [3].Various genes (*Saccharomyces cerevisiae* proteins (*STE12, STE5, STE11, STE7, STE20, STE18, STE4*), kinase suppressor of Sst1 (*KSS1*), and mitogen-activated protein kinase (*FUS3*)) are required for sensitivity to osmotin [12]. Osmotin increases its cytotoxic effect to susceptible fungi by initiating intercellular signal transduction pathways through STE7 genes in order to weaken the fungal cell wall [47]. After that the cascade of mitogen-activated protein kinase, which increases the defensive plasma membrane permeability, allows osmotin to enter the membrane, leading to trans-membrane pores which causes leakage in membrane and ultimately results in membrane rupture [3,11,45].

An experiment on spheroplast, fungi, and yeast (*Saccharomyces cerevisiae*) showed that the plasma membrane is the main target of osmotin protein [5]. In potato, late blight disease (*Phytophthora infestans*) was overcome through osmotin application and a large number of transgenic plants containing osmotin were successfully developed within a short span of time as compared to the cultivated potato [12].

Overexpression of osmotin protein has shown anti-fungal activities against basal rot (*Fusarium oxysporum*), late blight (*Phytophthora infestans*), fusarium head blight (*Fusarium graminearum*), wheat leaf rust (*Puccinia triticina*), and fruit rot (*Phytophthora capsici*) [7]. Tobacco osmotin also inhibits growth of green mold (*Trichoderma reesei*), red bread mold (*Neurospora crassa*), and candida (*Candida albicans*) [14].

Transgenic mulberry (*Morus indica*) plants harboring the osmotin gene induced resistance to different fungal infections (anthracnose: *Colletotrichum dematium*, bitter rot: *Colletotrichum gloeosporioides,* and crown rot: *Fusarium pallidoroseum*) by preventing infection in leaves to a few necrotic spots (10%), whereas in non-transgenic plants, 80–90% of leaves were covered with necrotic spots [4]. Similarly, transgenic soybean (*Glycine max*) leaves also exhibited resistance to powdery mildew (*Microsphaera diffusa*), Asian rust (*Phakopsorapa chyrhizi*), and brown spot (*Septoria glycines*) by showing negligible symptoms of diseases and maintaining chlorophyll pigment in leaves [35].

Transgenic plants of barley (*Hordeum vulgare*) infected with basal rot (*Fusarium oxysporum*) showed reduction in disease symptoms. Furthermore, no damage to DNA was observed in comet assay [10]. Transgenic kiwifruits (*Actinidia deliciosa*) showed resistance towards postharvest damage, improved shelf life and exhibited tolerance to artificial inoculation of fungal diseases (side rot: *Cadophora luteo-olivacea* and gray mold: *Botrytis cinerea*) [48]. However, in transgenic mustard plants (*Brassica juncea*), osmotin did not completely provide the resistance against blight (*Alterneria blight*), but delayed the appearance of disease symptoms [49]. In addition, tobacco osmotin combined with rice chitinase (*chi11*) effectively protected the transgenic tomato (*Solanum lycopersicum*) plants from basal rot disease (*Fusarium oxysporum*) [50] and transgenic rice plants from sheath blight disease (*Rhizoctonia solani*) [45,51].

Despite its broad range of antifungal activity, osmotin is still not effective against many strains of fungi such as *Macrophomina phaseolina* (charcoal rot)*,* mold (*Aspergillus parasiticus*), and kernel rot (*Aspergillus flavus*) [11].

### 5.2. Viral

Just like antifungal properties, osmotin also expressed tolerance to viral infections when accumulated in leaves of tobacco var Samsun NN through the hypersensitive response to tobacco mosaic virus (TMV) [52,53,54]. Moreover, osmotin-like proteins (OLPs) were also expressed in tomato infected with tomato (*Solanum lycopersicum*) planta macho viroid (TPMV) [55] and habanero pepper (*Capsicum chinense*) with pepper mild mottle virus (PMMoV) [56].

### 5.3. Bacterial

Osmotin does not have direct anti-bacterial properties and there are many bacterial strains, e.g., *Agrobacterium tumefaciens* that turn the host activity using cytokinins into their own favor. However, osmotin shows a close relationship to morphogenesis-related hormone cytokinins. It is assumed that osmotin might bind with cytokinins, causing deactivation of cytokinins which prevents the bacteria from causing tumors as cytokinin elimination prevents the spread of bacterial infection. This is an indirect role of osmotin (anti-bacterial) in addition to antifungal activity [11]. In an experiment, the overexpression of an osmotin-like protein (*CaOSM1*) in transgenic Arabidopsis (*Arabidopsis thaliana*) was observed, which showed an enhanced resistance to bacterial speck disease (*Pseudomonas syringae* pv. *Tomato*) by hypersensitive cell death, which prevented the proliferation of pathogens to healthy cells and increased accumulation of H_2_O_2_ which triggered the induction of different defense responses [47].

## 6. Osmotin-Like Proteins (OLPs)

Osmotin-like proteins (OLPs) belong to the pathogenesis-related (*PR-5*) protein family and can protect plants from different biotic and abiotic stresses [3]. OLPs also show tissue-specific expression. Under oxidative stress they are expressed in the meristematic region of the shoot apex and the quiescent (inactive) region of the root apex. Their activities were studied in root, stem, leaves, and flowers of lack nightshade (*Solanum nigrum*) and Arabidopsis (*Arabidopsis thaliana*), whereas in the root and shoot apex of cork oak (*Quercus suber*), in the flowers and fruit of tomato (*Solanum lycopersicum*), in grape ovaries [16], in developing oat (*Avena sativa*) and barley (*Hordeum vulgare*) seeds, in tobacco (*Nicotiana tabacum*) explants during flowering, and in grape (*Vitis vinifera*) berries during ripening [6]. Osmotin and thiamine-like proteins have also been isolated from sorghum, tobacco, wheat, barley, tomato, grape, banana, petunia, strawberry, eggplant, sweet pepper, soybean, sweet basil, and plumeria [57,58,59,60,61,62,63]. These osmotin-like proteins were also induced in trichomes and in the leaves of cadmium-stressed tobacco plants resulted by biotic and abiotic stresses [64], in potatoes (*Solanum tuberosum*) due to high atmospheric CO_2_ [65], as well as in petunia *(Petunia hybrida*) because of fungal infection, salicylic acid, aspirin, and wounding [59]. Many researchers have isolated, cloned, and characterized osmotin-like proteins from different plants and studied their overexpression-induced resistance to various biotic and abiotic stresses (Table 1).

Several osmotin-like proteins (OLPs) which showed a broad range of anti-fungal activity under in vitro conditions and in transgenic plants have been identified. The anti-fungal activity of osmotin-like proteins may be due to its structure. Its three-domain structure contains an acidic cleft between the I and II domains, which is involved in the catalytic activity of *β*(1,3)-glucan, which is an important component of fungi cell walls. Moreover, it is also recognized by a membrane receptor which initiates a pathway that results in accumulation of reactive oxygen species (ROS), that ultimately leads to cell death [66].

Apart from their anti-fungal properties, OLPs also have other important roles in plant physiological and developmental functions, such as flower formation, fruit ripening, antifreeze activity, and protection against osmotic stress, such as in strawberry (*Fragaria × ananassa*) where the expression level of *FaOLP2* increased to 2-fold in ripe fruit as compared to green fruit [67].

Transformation of the osmotin gene (Sind*OLP*) from black nightshade (*Solanum nigrum*) into sesame (*Sesamum indicum*) through *Agrobacterium tumefaciens* enabled transgenic plants to survive against various biotic and abiotic stresses (charcoal rot pathogen, oxidative stress, salinity, and drought). Transgenic plants activated many defense mechanisms that enabled them to increase root length, water content, chlorophyll, proline, and secondary metabolite content, improved ROS scavenging enzymes activity, and caused smaller stomatal aperture and less electrolyte leakage [8]. In addition, *Sn*OLP conferred drought tolerance in transgenic soybean (*Glycine max*) by increasing the net CO_2_ assimilation rate, maintaining higher respiration rate, stomatal conductance, and leaf water potential. However, a decrease in grain production and 100 grain weight in transgenic lines was observed, but was still higher than non-transgenic lines [58]. An osmotin-like protein (*ObTLP1*) from sweet basil (*Ocimum basilicum*) enhanced tolerance in transgenic Arabidopsis (*Arabidopsis thaliana*) from abiotic (salt, dehydration) and biotic (gray mold: *Botrytis cinerea*, white mold: *Scleretonia sclerotiorum*) stresses [68]. Osmotin gene (*TIOsm*) from five-minute grass (*Tripogon loliiformis*) induced abiotic stress tolerance (cold, drought, and salinity) in transgenic rice (*Oryza sativa*) by maintaining membrane integrity, growth, and improving survival rate [21].

However, development of stress resistance in plants requires interaction of multiple genes to induce tolerance; therefore, it is observed that another pathogenesis-related protein (PR-3) chitinase (*Chi11*) exhibits cross tolerance with OLPs. A study in tomato (*Solanum lycopersicum*) transgenes containing OLPs and chitinase (*Chi11*) genes confirms improved resistance to fungal, drought, and salt stress by improving vascular conductivity, total biomass, K^+^ content, relative water content, endochitinase activity, and chlorophyll fluorescence, respectively [26,69]. In transgenic peanut (*Arachis hypogea*) plants, a combination of PR genes (OLPs from *Solanum nigrum*: *SniOLP* and antifungal protein from *Raphanus sativus*: *Rs-AFP2*) improved resistance to late leaf spot disease by reducing the number and size of lesions [70].

### Role as Pharmaceutical Drug

Osmotin is similar to a naturally occurring human protein (adiponectin) in terms of functional and structural homology. Osmotin is a multifaced protein inducing tolerance in plants towards various biotic and abiotic stresses, whereas adiponectin is an anti-atherosclerotic and anti-diabetic protein that is decreased in obese patients. Both proteins have a role as anti-tumor proteins by suppressing *p53* gene and caspase enzyme activity [12]. Due to sequence homology of the osmotin receptor with the adiponectin receptor, it can be used as a pharmaceutical drug to treat diabetes, atherosclerosis, and obesity. Moreover, osmotin had no adverse effect on human embryonal kidney cells even at higher concentrations of 500 µg.mL^−1^ and did not exhibit any hemolytic activity (destruction of red blood cells). In fact, its antimicrobial property enables it to work against common food spoilage strains and pathogens (fungal) such as otitis and candidiasis [71].

## 7. Conclusions

Under biotic and abiotic stress conditions, many defense-related proteins are accumulated by plants and among them osmotin and its homologous proteins (OLPs) from the pathogenesis-related protein (PR) family 5 are considered as important due to their role in inducing stress resistance in plants. Thus, these proteins have been transformed successfully into economically important crops like tomato, rice, peanut, soybean, olive, kiwifruit, etc., through *Agrobacterium tumefaciens* and their mechanisms in initiating tolerance to various biotic (fungal and bacteria) and abiotic (drought, salinity, cold) stresses have been studied.

## Figures and Tables

**Table 1:** Overexpression of osmotin-like proteins (OLPs) confers stress tolerance in plants.

Crop	OLPs	Overexpression	Reference
Chili (*Capsicum sp.*)	*CaOSM1*	Induced resistance to bacterial and fungal pathogens	Hong et al. (2004) [6]
Soybean (*Glycine max*)	*GmOLPa*- acidic isoform	Induced in roots and leaves to protect plants from dehydration and salt stress	Onishi et al. (2006) [60]
Potato (*Solanum tuberosum*)	*OSM*	Improved plant resistance to salt stress	Aghaei et al. (2008) [34]
Soybean (*Glycine max*)	GmOLPa	Developed resistance in plant against salinity and drought	Tachi et al. (2009) [72]
Potato (*Solanum tuberosum*)	OSM-1	Overexpression induced resistance to late blight disease (*Phtyophthora infestans*)	EL-Komy et al. (2010) [73]
Sodom apple (*Calotropisprocera*)	*CpOSM*	Induced in latex and protect plants from bitter rot (*Colletotrichum gloeosporioides*) and red bread mold (*Neurospora sp*)	de Freitas et al. (2011) [57]
Black pepper (*Piper colubrinum*)	*PcOSM2*	Antifungal activity against wilt disease (*Phytophthora capsici*) and basal rot (*Fusarium oxysporum*)	Mani et al. (2012) [74]
Sodom apple (*Calotropisprocera*)	*CpOSM*	Initiated defense mechanism against root rot (*Fusarium solani*) spores	Ramos et al. (2015) [75]
Chili (*Capsicum sp*)	*CaOSM1*	Conferred tolerance to salt stress	Maurya et al. (2015) [76]
Cacao (*Theobroma cacao)*	*TcOsm1*	Inhibited growth of yeast and phytopathogenic fungi	Falcao et al. (2016) [66]
Rice (*Oryza sativa*)	*OsOSM1*	Induced in leaf sheath at booting stage and overexpression enhanced resistance to sheath blight disease	Xue et al. (2016) [77]
Syrian rue (*Peganum harmala*)	*OSM*	Induced resistance to salinity	Karam et al. (2016) [78]
Grapes (*Vitis vinifera*)	*VvOSM1*	Overexpression improved salinity tolerance	Saleh and Alshehada, (2018) [79]
Sugar beet (*Beta vulgaris*)	*OSM*	Overexpressed under PEG induced drought stress	Youssef et al., (2018) [80]
Eucalyptus (*E. tereticornis and E. camaldulensis*)	*OSM34*	Protected nursery plants from induced water stress	Amrutha et al. (2019) [81]
Chinese ginseng (*Panax notoginseng*)	*PnoLP1*	Involved in defense response against *Fusarium solani*	Zhao et al., (2020) [82]
